# Challenges of Providing Palliative Care to a Patient With Learning Disability: A Case Study From United Kingdom General Practice

**DOI:** 10.7759/cureus.14240

**Published:** 2021-04-01

**Authors:** Lubna Rauf, Khalid Bashir

**Affiliations:** 1 Clinical Education, Qatar College of Medicine, Doha, QAT

**Keywords:** palliative and supportive care, end of life and hospice care, holistic pain management, integrated delivery of health care, team based end of life care, consent and capacity in uk law, caring for people with learning difficulties, uk mental capacity act

## Abstract

Palliative care is a complex and challenging field in the healthcare profession. In the United Kingdom (UK), palliative care provision is part of everyday work for General Practitioners (GPs). The UK General Practice Curriculum includes palliative care as a core competency to be achieved to become a fully certified GP/family physician. The various stages of a patient’s journey from getting a life-limiting diagnosis to breaking bad news, and dealing with the treatments and resulting complications need active involvement from the patient and their loved ones with healthcare professionals at all times. It becomes more challenging if the patient has impaired mental capacity and cannot make his independent decisions as a result. The interplay of patient’s wishes, the wishes of immediate relatives, the law of the land and clinician’s role in becoming an advocate to safeguard patient’s best interest has significant implications for all stakeholders and far-reaching consequences. This case study elaborates on some of the challenges in the delivery of palliative care in a complex situation and provides knowledge base to bridge those flaws.

## Introduction

This case encompasses issues around end-of-life care in a scenario of patient with learning difficulties. It highlights the legal and ethical responsibilities of healthcare professionals according to the United Kingdom’s (UK) law in such cases. In this case, the healthcare professionals assumed the parents’ decisions on patient’s behalf to be valid and correct without any probing into patient’s personal beliefs or parents’ level of understanding. This is against the UK legal and ethical framework of caring for the people who lack capacity. It resulted in unsatisfactory outcomes for the patient during his diagnosis of a life-limiting illness and its management. It is absolutely necessary for health care professionals to understand and implement Mental Capacity Act to safeguard patients’ best interests. The need for holistic management of such patients by multi-professional health care workers working together is been put in the spotlight. The guidelines to inform and empower health care providers to achieve the best possible care in such scenarios have been sign-posted.

## Case presentation

CJ was a 49-year-old man. He belonged to a family of Jehova’s witnesses. He had undiagnosed learning difficulties (LD) due to which he was never able to lead an independent life. He lived with his parents. No other agencies were involved in his care and he was not seen by the General Practitioner (GP) for years. He was brought in to be seen by the GP for a routine appointment by his father. He was frail and looked unwell but did not seem to be in any discomfort. Examination revealed a rectal mass and palpable inguinal lymph nodes. An urgent referral to the colorectal surgeons was made and blood tests were performed. He was found to be profoundly anaemic. Due to the parents’ belief, no blood products were accepted for CJ. The hospital also offered an inpatient care which was refused by his parents.

After being seen by the consultant in the hospital, he had an MRI scan which revealed a locally advanced rectal tumour. A de-functional colostomy was performed for palliative purposes by the surgical team due to mechanical bowel obstruction secondary to tumour size and location. The specialist multidisciplinary team (MDT) decided to go ahead with aggressive chemotherapy to stop further tumour spread.

Once the patient was discharged back home, the district nurses were involved in providing stoma care to the patient. During this time, he had multiple chemotherapy sessions and two incidences of neutropaenic sepsis. A follow-up MRI scan few months later found metastasis to liver. Patient was commenced on palliative care pathway with the aim to reduce discomfort.

The patient’s health was deteriorating slowly. He was started on nutritional support and was frequently assessed by the community palliative care team. They were making regular contact to assess pressure areas, provide any stoma care or dressings if needed and helped parents out with his basic needs and medication. There was a little hospice or GP involvement during that time.

His end-of-life care was carried out in his parents’ house as they did not allow him to go elsewhere. Due to the accessibility issues and the size, the house and CJ’s room was unsuitable to deliver care in the best possible manner. The bedroom where CJ spent his time could only accommodate a bed where he would lie all day. There was no space for a chair or a recliner for him to sit to prevent bedsores. The parents did not allow any hoisting equipment or changes to their existing bathroom and showering facilities to accommodate CJ’s needs. It resulted in poor hygiene and lack of dignity for CJ towards his end of life. The discussions by the community healthcare team about these issues with parents were brief and when parents denied all help, colleagues like GPs and palliative care consultants in hospice were not involved to further explore the reasons of parents’ reluctance to accept help which might have enabled in improved outcomes for CJ. There was no decision around patient’s resuscitation status as parents refused this discussion on many occasions as well.

He had multiple visits to the emergency department (ED) during this time which could have been avoided. CJ used to be taken to hospital in an ambulance to the ED where he used to wait for a clinician on the trollies for hours. He used to get unnecessary investigations and intravenous cannulation.

CJ died in his own home after 16 months of his diagnosis.

## Discussion

The principal issue in CJ’s management is lack of exploration of his mental capacity and ability to consent. It was assumed that he was in agreement with whatever parents decided for him and he was not able to make any decisions on his own. There seems to be little evidence available that anyone tried to ascertain what he wanted and whether his beliefs were different from his parents. There had been no mention of assessment of his capacity at any point. There was no documentation of the fact whether he knew what was wrong with him and what was supposed to happen next. If a recognized model of breaking bad news like SPIKES had been used, the barrier of mental capacity would have been identified straightaway and further discussions would have been instigated by involving specialist palliative care team to help ascertain the level of capacity [1]. It would have enhanced his decisions at all levels of his care and his autonomy would have been safeguarded.

Effective communication seemed to have lacked in this case in all steps of management. Every attempt should have been made to make the patient aware of their diagnosis and the steps that followed on. A CLASS protocol is a good guide to enhance the ability of communication with consideration to emotions. This protocol stresses upon Context (C) of a medical interview with active listening (L), to acknowledge (A) emotion, to give a management strategy (S), with summary (S) and closure [2]. If CJ was found to have little understanding of his issues and his limited mental capacity had been objectively established, his parents could also have understood things better and could have engaged in meaningful discussions about their son if such a protocol would have been used.

Not only did the healthcare professionals fail to ascertain his understanding of the diagnosis, a formal assessment and documentation of his mental capacity around specific issues of his care was not done at multiple levels of care. It resulted in failure to facilitate necessary help based on a legal and ethical framework of care provision.

Assessment of capacity around the decision of refusing transfusion and initial admission to hospital was crucial. If it was established that he lacked capacity by employing the Mental Capacity Act (MCA) 2005, his best interests would have been safeguarded by involving a third party (Independent Mental Capacity Advocacy [IMCA]) who could have helped to achieve better care of patient’s unmet needs [3]. This was more important specifically for this case as parents seemed to have little understanding of issues as well.

The parents' understanding of the rationale of their decisions was never explored. The family’s decisions should be always acknowledged and respected wherever possible [4]. However, in this case, the parents were making decisions for their son in absence of any guidance and support from healthcare professionals which resulted in many decisions in conflict of patient's best interest. The MCA could be used to not only assess patient’s capacity to make individual decisions around his care but it could also have aided in making sound and lawful decisions in the best interest of the patient [3].

During CJ’s second admission in hospital with neutropaenic sepsis, he was seen by the lead palliative care nurse due to uncontrollable pain and discomfort. The initial pain assessment was made using the Brief Pain Inventory [5]. However, “holistic pain management” was not mentioned which could have encompassed any psychological or spiritual aspects of his discomfort or pain. Because CJ was unable to express fully how he felt, it was more vital to establish his pain according to a recognised tool which could tell the healthcare professionals about him as an individual. Scottish Intercollegiate Guidelines Network (SIGN) have effective and robust guidelines for holistic pain management [6].

As Melzack et al. explain in "The Challenge of Pain", pain is not a single sensation, but is defined as category of complex experiences [7]. As a result, only treating physical cause of CJ’s pain was not going to help. The unresolved spiritual or psychological issues will not only keep the pain threshold very low, it can also reduce the efficacy of the analgesia used.

As the caring issues became more complex, the involvement of IMCA would have given the whole process clarity and necessary transparency which is vital in ethically challenging decisions [3]. IMCA is a legal safeguard for people who lack the capacity to make specific and important decisions. IMCA can act on behalf of a patient when patient cannot make their own decisions. Their purpose is to have a legal representation of patients’ best interest in absence of relatives or if relatives have little or no understanding of the issues surrounding their care.

When CJ was placed on end-of-life care pathway, he was still subjected to frequent investigations which had little to contribute in his management and resulted in unnecessary distress of phlebotomy, sometimes even under partial restraint. There was no cohesion among the primary and secondary care provision as there was no communication between the two parties. The parents took him to the ED every time he had a complaint because they did not know any other channels of help and support. There was a mismatch between the actual needs of the patient and parents’ perception of his illness. No time was invested in aligning those to make things better for CJ.

Providing treatment and care towards the end of life often involves decisions that are clinically complex and often emotionally distressing. Some decisions involve ethical dilemmas and uncertainties about the law that further complicates the decision-making process. According to General Medical Council (GMC), this requires working of all health care providers in unison to minimize the flaws and remain within the ethical and legal boundaries [8].

## Conclusions

Palliative care is a complex undertaking that is only achievable by a joint effort by all care providers. The leadership lies within the hands of the health professionals who can play a pivotal role in opening the channels of communication between the patients, the carers and all healthcare providers. The joint work can formalise care according to the best practice guidelines. It will result in the consistency of care and will improve experience of care for the patient, carers and staff. The accessibility of primary care records in secondary care and vice versa can minimise duplication and result in better use of resources. Healthcare professionals must be competent and confident in the application of Mental Capacity Act in the formal assessment of mental capacity. The MCA must be an overarching principle in initiating and continuing healthcare for people with learning disabilities. The contemporaneous documentation of every step of management is of paramount importance. In challenging cases, it is of utmost value to involve specialist palliative care and community teams health care teams from initial stages who can help with their knowledge and resources to implement best practice. The psychological issues and issues around complex care needs are addressed in the best way by exploring the understanding of patient and carers and aligning the goals of all concerned parties. The rights and best interest of the patient must be upheld through all circumstances.

The GMC guidance of Treatment and Care Towards the End of Life can be used as a gold standard document to care for the dying which is in line with the UK legal and ethical framework. The GMC document recommends patients' comfort supported mainly by the caregivers and GPs. It is best to avoid unnecessary and invasive treatments such as antibiotics, cardiopulmonary resuscitation (CPR), renal dialysis, parenteral nutrition, fluids and mechanical ventilation. The document also elaborates on managing patients who lack capacity by an appointment of an independent attorney or legal proxy, and provide the treatment in the best interest of the patient. It is also recommended that providing palliative care for the patients who are unable to consent should be done through a multi-disciplinary approach involving GPs, local care homes, ambulance service, local health authority and voluntary support workers.

## References

[1] https://www.themdu.com/guidance-and-advice/guides/breaking-bad-news.

[2] https://www.mdanderson.org/documents/education-training/icare/pocketguide-texttabscombined-oct2014final.pdf.

[3] https://www.nhs.uk/conditions/social-care-and-support-guide/making-decisions-for-someone-else/mental-capacity-act/.

[4] https://www.nice.org.uk/guidance/cg138/ifp/chapter/Your-relationships-with-healthcare-professionals.

[5] https://www.britishpainsociety.org/static/uploads/resources/files/Outcome_Measures_January_2019.pdf.

[6] Cormie PJ, Nairn M, Welsh J (2008). Control of pain in adults with cancer: summary of SIGN guidelines. BMJ.

[7] Melzack R, Wall P (1982). The Challenge of Pain. https://research.monash.edu/en/publications/the-challenge-of-pain-by-r-melzack-amp-p-wall-melbourne-penguin-b.

[8] https://www.gmc-uk.org/-/media/documents/treatment-and-care-towards-the-end-of-life---english-1015_pdf-48902105.pdf.

